# Mental chronometry in the pocket? Timing accuracy of web applications on touchscreen and keyboard devices

**DOI:** 10.3758/s13428-019-01321-2

**Published:** 2019-12-10

**Authors:** Thomas Pronk, Reinout W. Wiers, Bert Molenkamp, Jaap Murre

**Affiliations:** 1 Department of Psychology, Faculty of Social and Behavioural Sciences, University of Amsterdam, Amsterdam, The Netherlands; 2 Behavioural Science Lab, Faculty of Social and Behavioural Sciences, University of Amsterdam, Amsterdam, The Netherlands

**Keywords:** Timing Accuracy, Online research, Individual differences, Smartphones, Laptops, Javascript, Response time

## Abstract

Web applications can implement procedures for studying the speed of mental processes (mental chronometry) and can be administered via web browsers on most commodity desktops, laptops, smartphones, and tablets. This approach to conducting mental chronometry offers various opportunities, such as increased scale, ease of data collection, and access to specific samples. However, validity and reliability may be threatened by less accurate timing than specialized software and hardware can offer. We examined how accurately web applications time stimuli and register response times (RTs) on commodity touchscreen and keyboard devices running a range of popular web browsers. Additionally, we explored the accuracy of a range of technical innovations for timing stimuli, presenting stimuli, and estimating stimulus duration. The results offer some guidelines as to what methods may be most accurate and what mental chronometry paradigms may suitably be administered via web applications. In controlled circumstances, as can be realized in a lab setting, very accurate stimulus timing and moderately accurate RT measurements could be achieved on both touchscreen and keyboard devices, though RTs were consistently overestimated. In uncontrolled circumstances, such as researchers may encounter online, stimulus presentation may be less accurate, especially when brief durations are requested (of up to 100 ms). Differences in RT overestimation between devices might not substantially affect the reliability with which group differences can be found, but they may affect reliability for individual differences. In the latter case, measurement via absolute RTs can be more affected than measurement via relative RTs (i.e., differences in a participant’s RTs between conditions).

## Introduction

During the past decade, touchscreen devices (i.e., smartphones and tablets) have surpassed keyboard devices (i.e., desktops and laptops) to become the most frequently used devices for browsing on the internet (StatCounter, 2016). Web browsers offer platforms, based on widely supported open standards (such as HTML, CSS, and JavaScript) for deploying web applications to both touchscreen and keyboard devices. Hence, a research paradigm implemented as a web application can be deployed in the lab as well as on any commodity device, while also having the benefits of being based on open and durable standards. It has become more common to employ web applications for questionnaire research, but less so for mental chronometry (i.e., study of the speed of mental processes). This is an important limitation, because psychological research increasingly employs mental chronometry to indirectly assess psychological constructs, which has been argued to help the validity of assessments by reducing the influence of socially desirable answering (De Houwer, Teige-Mocigemba, Spruyt, & Moors, 2009; Greenwald, Poehlman, Uhlmann, & Banaji, 2009). If mental chronometry can reliably be conducted on touchscreen devices, this offers the opportunity to conduct such research on a wider range of samples, such as the inhabitants of emerging economies (Pew Research Center, 2016), and in a wider range of contexts, such as naturalistic settings (Torous, Friedman, & Keshavan, 2014), than previously had been feasible.

One reason that assessments of mental chronometry on commodity devices via web applications have been limited is doubt about whether commodity devices have sufficiently accurate timing capabilities (Plant & Quinlan, 2013; van Steenbergen & Bocanegra, 2016). A range of studies have assessed the timing accuracy of web applications, but, to the best of our knowledge, only with keyboard devices. We make a general assessment of the technical capabilities of keyboard and touchscreen devices for mental chronometry paradigms in which a single static stimulus is presented to which a single response is registered. Such paradigms may require that stimuli are accurately presented for a specified duration and response times (RTs) are accurately measured. Factors that determine what level of accuracy is achieved, include the capabilities of the device, operating system (OS), web browser, and methods for timing stimulus presentation and registering responses. Factors that determine what level of accuracy is required, include the demands of the particular paradigm under consideration, the degree to which systematic differences in accuracy across devices can be confounding variables, and to what extent these can be compensated for by increasing the number of trials or participants.

With regard to stimulus presentation, web applications may occasionally realize shorter or longer durations than were requested (Barnhoorn, Haasnoot, Bocanegra, & van Steenbergen, 2015; Garaizar & Reips, 2018; Garaizar, Vadillo, & López-de-Ipiña, 2014a; Reimers & Stewart, 2015; Schmidt, 2001). Computer screens refresh with a constant frequency and presentation durations are typically counted in frames. The most common refresh rate is 60 Hz, so that a frame lasts about 16.67 ms. Timing errors can be expressed as the *frame difference*, which is the number of frames realized minus the number of frames requested (called *missed frames* by Garaizar, Vadillo, & López-de-Ipiña, 2014a; Garaizar, Vadillo, López-De-Ipiña, & Matute, 2014b). We presuppose that a frame difference of one (e.g., 16.67 vs. 33.33 ms) is problematic for mental chronometry paradigms in which stimuli are presented very briefly or very precisely, such as in Posner tasks (Posner, 1980), stop signal tasks (Logan, Cowan, & Davis, 1984), and tasks using very briefly presented masked stimuli (Marcel, 1983). For longer durations, such as 250 ms, frame differences may be less problematic, as long as the realized duration does not differ too greatly from the requested duration (e.g., 266.67 ms may be an acceptable deviation, but 350 ms may not be).

With regard to RT measurement, research has indicated a noisy overestimation of RTs, with the mean and variance of overestimations varying across devices and browsers (Neath, Earle, Hallett, & Surprenant, 2011; Reimers & Stewart, 2015). In simulation studies, nonsystematic overestimation of RTs has generally been modeled as uniform distributions ranging up to 18 ms (Damian, 2010), 70 ms (Reimers & Stewart, 2015), or 90 ms (Brand & Bradley, 2012; Vadillo & Garaizar, 2016). Such RT overestimation was generally found to have a modest impact on a range of parameter estimation methods and designs, especially when scoring a task by subtracting RTs of a participant between two or more conditions. Such subtracted scores will henceforth be referred to as *relative RTs*. However, systematic differences in RT overestimation between devices may form a confound when device preference systematically varies with a trait under study, measured via absolute RTs (Reimers & Stewart, 2015). Devices may also quantize RTs into supramillisecond resolutions (Reimers & Stewart, 2015). Even in this case, simulations have revealed that resolutions of up to 32 ms may have little impact on reliability (Ulricht & Giray, 1989). An important limitation to these studies is that they have generally examined the reliability with which group differences can be found, but did not explicitly address the reliability with which individual differences can be found. Studies of individual differences may require more reliable measures than studies of group differences (Hedge, Powell, & Sumner, 2018).

Web applications offer different methods for timing and presenting stimuli, as well as registering responses. Recently, particular methods for optimizing timing accuracy have been introduced and examined. These include three methods with which a stimulus can be presented, based on manipulating the (1) opacity or (2) background-color Cascading Style Sheet (CSS) properties of a Hypertext Markup Language (HTML) element (Garaizar & Reips, 2018), and (3) drawing to a canvas element (Garaizar, Vadillo, & López-de-Ipiña, 2014a). The onset and offset of stimuli can be timed via requestAnimationFrame (rAF; Barnhoorn et al., 2015; Garaizar & Reips, 2018; Garaizar, Vadillo, & López-de-Ipiña, 2014a) for all three presentation methods listed above. Additionally, opacity and background-position presentation methods can be timed via CSS animations (Garaizar & Reips, 2018). Internal chronometry (i.e., measuring stimulus presentation and response registration using only the means available to the web application) may be used to improve timing accuracy while a task is being administered or to assess accuracy afterward (Anwyl-Irvine, Massonnié, Flitton, Kirkham, & Evershed, 2019; Barnhoorn et al., 2015; Garaizar & Reips, 2018).

In two experiments, we examined the accuracy of stimulus presentation (Exp. 1) and the accuracy of RT measurement (Exp. 2) via external chronometry (i.e., measuring stimulus presentation via a brightness sensor and generating responses via a solenoid). Accuracy was examined on ten combinations of devices and browsers, formed by two touchscreen and two keyboard devices, each running a different OS, and two or three browsers per device. Technical capabilities were evaluated for two research settings: a lab setting, in which the device and browser could be controlled, and a web setting, in which they could not. For the former setting, the most accurate devices and browsers were evaluated, and for the latter, we evaluated variation across devices and browsers. In Experiment 1, the accuracy of each of the presentation and timing methods listed above was first assessed on the basis of the proportion of trials in which the realized stimulus duration was exactly the number of frames that was requested. Next, the most accurate method was further examined in terms of the distribution of frame differences, reporting on different methods if they produced notably different patterns of results. We compared the accuracy with which stimuli were presented for both brief and long durations—across devices and browsers—in order to assess the degree to which mental chronometry paradigms may be affected that require very brief or precise stimulus durations.

In Experiment 2 we assessed how accurately web applications can measure RTs across devices and browsers. RT overestimations were expected to vary substantially, both within and between devices and browsers. To assess how well RT overestimations represented simulation assumptions, we examined distributions across devices, paying particular attention to the presence of any quantization. As in Experiment 1, only the most accurate method of timing and presenting stimuli was considered for further investigation; we have reported on different methods if they produced notably different patterns of results. Informed by the findings of Experiment 2, we conducted a set of simulations to quantify the impact of the accuracy of RT measurements on measurement reliability. In contrast with prior modeling, our simulation did not examine the reliability with which differences between groups or conditions can be detected, but the reliability with which individual differences can be measured.

Summarizing, the present study describes how accurately web applications on keyboard and touchscreen devices can present stimuli and register the responses. For stimulus presentation, we examined the presence and magnitude of timing errors in terms of frame differences. For RT measurement, we examined the accuracy with which RTs are measured, the distribution of RT overestimations, and how these might affect the reliability with which individual differences can be measured. Exploratively, we examined a set of methods for improving timing accuracy based on different approaches to timing stimuli, presenting them, and measuring internal chronometry, so as to assess the most accurate method on offer for modern devices and browsers.

## Method

### Devices

Table 1 lists the characteristics of the devices used in the study. We selected one laptop for each of two popular keyboard OSs (MacOS and Windows) and one smartphone for each of two popular touchscreen OSs (Android and iOS). Below, these devices will be referred to via their OS. All four devices were normally in use as commodity devices by colleagues of the first author. We selected web browsers that were widely supported for each device: Chrome 71.0.3578.99 and Firefox 64.0.2/14.0 for all OSs, and Safari 12 for MacOS and iOS. These browsers were selected for being relatively popular (StatCounter, 2018), still being actively developed at the time of the study, and each being based on a different browser engine—namely Blink, Gecko, and WebKit, respectively. A device variable not included in our experiments was device load, because this seems to have only minor effects on modern devices (Barnhoorn et al., 2015; Pinet et al., 2016) and is difficult to manipulate systematically (and reproducibly) on touchscreen OSs.

**Table 1:** Model, model year and month, model number, and operating system (OS) of each device

Model	Year and Month	Model Number	OS
MacBook Pro	October 2016	A1398, EMC 2910	MacOS 10.13.2
ASUS laptop	February 2016	R301LA-FN218T	Windows 10.0.17134.523
Samsung Galaxy S7	March 2016	SM-G935F	Android 8.0.0
iPhone 6S	September 2015	MKU62ZD/A	iOS 12.1.2

### Design

During the stimulus presentation experiment, stimuli were presented for intervals of 16.67, 50, 100, 250, and 500 ms. As each device had a refresh rate of 60 Hz, these intervals corresponded to 1, 3, 6, 15, and 30 frames. The stimulus was a white square on a black background. The presentation experiment consisted of 120 sequences, in which each of the five combinations of timing and presentation methods was first prepared by adding the relevant HTML element to the web page, followed by one trial with each of the five intervals, followed by removing the HTML element. Each sequence was administered in one unique order, out of the 120 possible orders in which the five intervals could be arranged. Each sequence and interval was demarcated by 400 ms of background, followed by 400 ms of an intertrial stimulus presented via a separate HTML element, followed by 400 ms of background. Because the intertrial stimulus was reliably presented for at least 200 ms across all devices, it could be used to match the individual trials of each sequence in terms of both internal and external measures. During the RT experiment, a stimulus was presented until a response had been registered. For each of the five presentation methods, a sequence of 300 RT intervals was generated, consisting of each whole number of ms in the range 150 to 449 in a pseudorandom order. Each interval was distinguished by 1,200 ms of background and each sequence was demarcated by 5,000 ms of background.

### Measures

#### Stimulus presentation and timing

Three presentation methods were compared, in which stimulus onset and offset were realized by (1) manipulation of the opacity CSS attribute of a DIV element by changing it from 0 to 1 or from 1 to 0; (2) manipulation of the background position of a DIV element by having a picture shift position such that a white or black part is presented; or (3) drawing a white or black rectangle on a canvas element. Two timing methods were compared, in which the stimulus onset and offset were (1) timed via CSS animations that manipulated the appropriate CSS properties, or (2) timed by having a call from rAF manipulate the appropriate CSS properties or draw to a canvas element, after a fixed number of frames. All manipulations were programmed via JavaScript, using the jQuery 3.31 library. The HTML, CSS, and JavaScript were configured according to the best practices recommended in previous timing research (Garaizar & Reips, 2018): Pictures were preloaded, each HTML element was laid out using absolute positioning in a separate layer, and the CSS properties used for presenting stimuli were marked with the will-change property.

#### Internal chronometry

In Experiment 1, for all stimulus timing methods, four internal estimates of stimulus duration were compared. These measures, described further in the online supplement, were based on *rAF before created*, *rAF before called*, *rAF after created*, and *rAF after called*. For timing via CSS animations, two additional estimates of stimulus duration were added, based on *animationstart/animationend created* and *animationstart/animationend called*. In Experiment 2, RT was internally measured as the time passed between stimulus onset and a keyboard or touchscreen response. For the moment of stimulus onset, the most accurate measure as found in Experiment 1 was selected. For the moment of response, two measures were compared, based on when a KeyboardEvent and TouchEvent were created or called.

#### External chronometry

The stimuli were detected via an optical sensor aimed at the top left of the device screen, which sampled luminance with a frequency of 3000 Hz. Sensors were calibrated via an Agilent 54624A oscilloscope (see the study protocol at the accompanying Open Science Foundation [OSF] repository). The signals were recorded via a dedicated computer running in-house-developed software (Molenkamp, 2019). Stimulus onset and offset were defined as brightness increasing above and decreasing below 50% of maximum screen brightness, respectively.

In Experiment 1, the realized stimulus duration was measured as the number of frames that passed between stimulus onset and offset. During data preprocessing, a range of checks were performed on the stimulus sensor data to verify whether detection of the stimulus duration was accurate—for instance, by verifying whether durations were quantized in multiples of 16.67 and whether the intertrial and intersequence intervals had plausible durations. When a critical stimulus was not presented, a black screen of about 800 ms should occur (twice the 400 ms background), followed by an intertrial stimulus of about 400 ms. See the analysis scripts at the accompanying OSF repository for more details.

In Experiment 2, an Arduino Leonardo microcontroller received a signal on stimulus onset, waited the number of milliseconds specified by each RT interval, and then sent a signal to trigger a solenoid. The solenoid was aimed at a touch-sensitive HTML element positioned at the bottom right of the screen of touchscreen devices and at the Q key of keyboard devices. After triggering, the solenoid consistently took 11 ms to go down and trigger a second optical sensor. The second sensor was positioned just above the point at which the solenoid would come in touch with the touchscreen or keyboard. The time between detecting a stimulus via the stimulus sensor and the solenoid touching the touchscreen or keyboard was considered the actual RT. Each interval between detecting stimulus onset and triggering the solenoid was reduced by 11 ms to correct for the solenoid delay.

### Procedure

For each combination of device, browser, and experiment, the device was first prepared by installing updates to the OSs and web browsers. The OSs of the MacOS and iOS devices were not updated, as their owners expressed concerns with the stability of the most recent OS versions at the time of the study. Screen brightness was set to maximum, and screensavers and notifications were disabled. Next, for each browser, the browser and experiment software were loaded, a minute was waited (to allow background processes to reach a stationary level), and the experiment was started. Per combination of device and browser, Experiment 1 took about 80 min, and Experiment 2 took about 40 min.

## Results

### Experiment 1

#### Accuracy per timing and presentation method

Table 2 shows the percentages of trials in which the realized duration was exactly as requested (i.e., the frame difference was 0) for each combination of device, browser, timing, and presentation method. To compare timing and presentation methods, we performed two-proportion *z* tests on all pairs of percentages collapsed across devices and browsers. A Holm–Bonferroni multiple-comparison correction was applied, with a family-wise alpha of .05, as was the case for all pairwise comparisons reported below. All differences were statistically significant except for rAF background versus rAF canvas; timing via rAF was more accurate than timing via CSS, regardless of the presentation method. When timed via rAF, presentation was more accurate via opacity (81.0%) than via background position (76.3%) or canvas (75.5%). When timed via CSS, presentation was more accurate via background position (23.7%) than via opacity (17.6%).

**Table 2:** Percentages of trials in which the realized duration was exactly as requested, per device, browser, timing method, and presentation method

OS	Web Browser	CSS		rAF		
		Background	Opacity	Background	Canvas	Opacity
Android	Chrome	32.3	30.2	64.0	60.7	78.8
Android	Firefox	8.2	8.3	42.5	64.7	82.7
iOS	Chrome	0.0	0.0	100.0	100.0	100.0
iOS	Firefox	0.0	0.2	100.0	99.8	100.0
iOS	Safari	0.2	0.3	100.0	99.8	100.0
MacOS	Chrome	55.7	50.0	59.3	55.0	51.5
MacOS	Firefox	19.8	3.5	61.7	53.0	61.7
MacOS	Safari	22.2	19.8	63.2	60.8	63.8
Windows	Chrome	59.3	63.7	100.0	100.0	99.8
Windows	Firefox	39.3	0.0	72.3	61.7	72.0

CSS, Cascading Style Sheet; rAF, requestAnimationFrame.

#### Accuracy per device and browser

We selected the most accurate timing and presentation method (rAF opacity) and made pairwise comparisons of all devices and browsers via two-proportion *z* tests. All differences were statistically significant, except for the following: between any of the iOS browsers and Windows Chrome, between any of the Android browsers and Windows Firefox, between any of the MacOS browsers, and between Windows Firefox and either MacOS Firefox or MacOS Safari. Hence, iOS could be considered very accurate, Android moderately accurate, and MacOS not accurate. The accuracy of Windows depended on the browser, with Chrome being very accurate, but Firefox being moderately to not accurate.

#### Accuracy of internal duration measures

We examined to what degree presentation duration could be estimated via internal chronometry, using four rAF-based measures and two animation-based measures. The distributions of rAF-based measures were strongly quantized, with a resolution of 16.67 ms, whereas the distributions of the animation-based measures were moderately so. To examine how accurately internal chronometry could predict realized duration, we quantized internally estimated durations into frames and calculated the percentage of trials in which the realized duration was exactly the same as that estimated via internal measures (i.e., the frame difference was 0). For all devices, browsers, timing, and presentation methods except for MacOS Safari, rAF-based were more accurate than animation-based duration measures. Collapsed across devices and browsers, each of the four rAF-based measures was correct on 75.8% to 76.4% of trials, whereas the animation-based measures were correct on only 34.8% to 34.9%. Two-proportion *z* tests on all pairs of percentages revealed significant differences between rAF-based and animation-based measures, but not within the rAF-based and animation-based measures. Since rAF-based measures were more accurate than animation-based measures, these were selected for further analysis. Because each of the four rAF-based measures was similarly accurate, *rAF before created* was selected for further analysis.

Table 3 shows the percentages of trials in which the frame differences were 0 per device, browser, timing method, and presentation method. When timing via rAF, estimating stimulus durations via internal chronometry was approximately as accurate as waiting a fixed number of frames was at achieving accurate stimulus durations (all *p* values for the *z* tests on percentages were ≥ .05). When timing via CSS, estimating stimulus durations via internal chronometry was more accurate than CSS animations were at achieving accurate stimulus durations (all *p* values were < .001).

**Table 3:** Percentages of trials for which the realized duration was exactly the duration measured internally via *rAF before created* timestamps quantized into frames

OS	Web Browser	CSS		rAF		
		Background	Opacity	Background	Canvas	Opacity
Android	Chrome	58.3	70.7	64.0	60.7	78.8
Android	Firefox	53.5	80.7	45.5	64.5	82.5
iOS	Chrome	93.2	97.5	100.0	100.0	100.0
iOS	Firefox	94.5	95.2	100.0	99.8	100.0
iOS	Safari	93.5	94.3	100.0	99.8	100.0
MacOS	Chrome	56.3	50.2	59.5	55.0	51.5
MacOS	Firefox	60.7	64.8	61.7	53.0	61.7
MacOS	Safari	30.5	24.2	63.2	60.8	63.8
Windows	Chrome	99.8	99.5	100.0	100.0	99.8
Windows	Firefox	60.3	84.0	72.3	61.7	72.0

CSS, Cascading Style Sheet; rAF, requestAnimationFrame.

#### Magnitude of frame differences

**Fig. 1:**
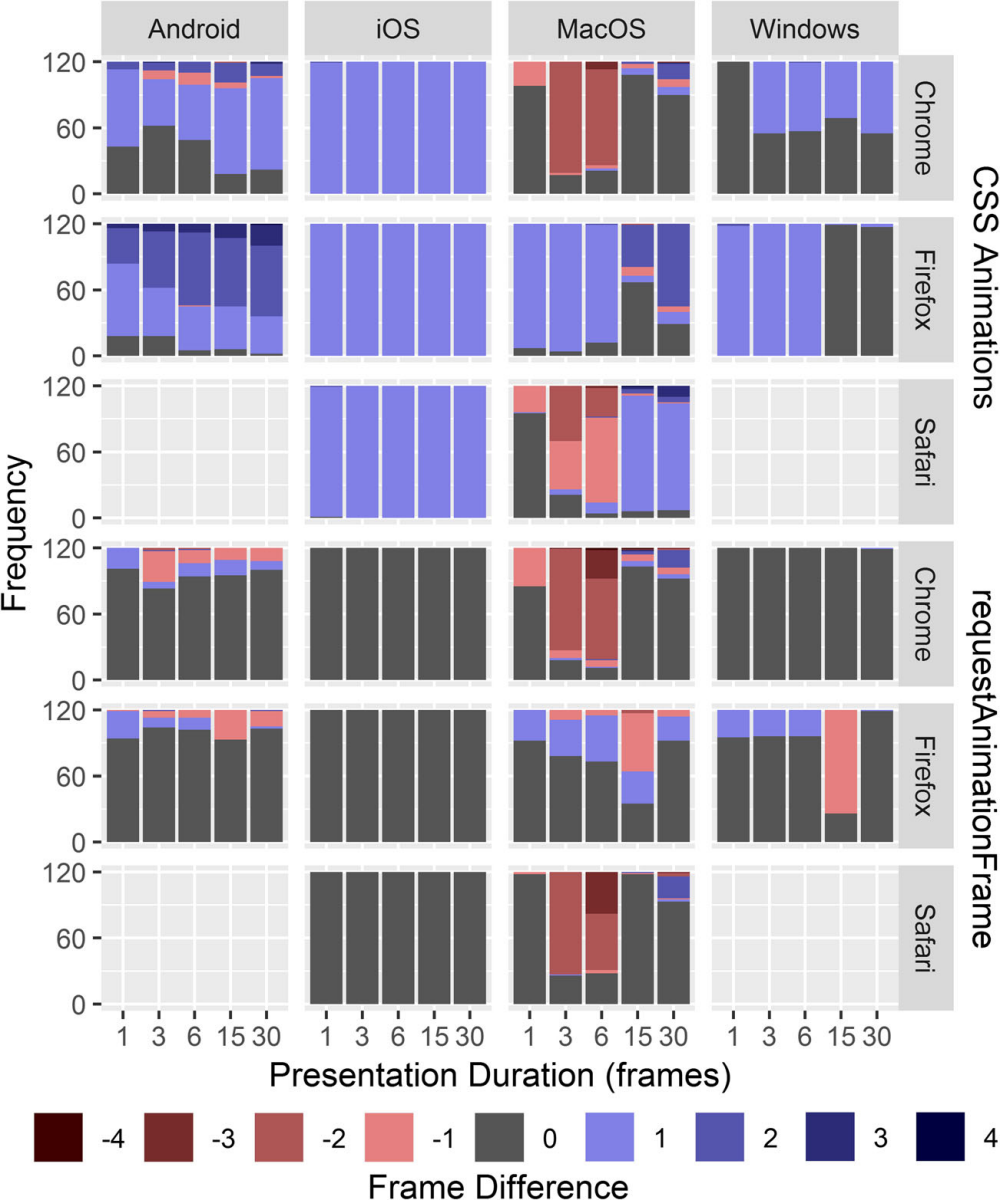
Stacked bar charts of the frequency with which frame differences ranged from -4 to 4 per device, browser, and duration interval, for presentation via background position with timing via CSS animations, and presentation via opacity with timing via requestAnimationFrame.

The distributions of frame differences were consistent across presentation methods within the rAF- and animation-based timing methods, but showed pronounced differences between timing methods. Therefore, we report on the most accurate presentation method for timing via rAF (opacity) and for timing via CSS (background color). There was variation in the sizes and signs of frame differences across devices, browsers, presentation methods, and intervals (Fig. 1). Both iOS and Windows realized almost all stimulus durations within one frame of the requested duration, as were stimuli timed via rAF on Android and MacOS Firefox. Timing via CSS yielded realized durations that were almost all consistently one frame longer than expected on iOS, as was the case on MacOS Firefox and Windows Firefox for one-, three-, and six-frame intervals. Finally, note that stimuli requested for three- or six-frame intervals were frequently presented too briefly on MacOS Chrome and MacOS Safari. In fact, the majority of three-frame intervals were presented for only a single frame.

### Experiment 2

#### Mean RT overestimation

For RT measurements, only trials presenting stimuli via the most accurate timing method (rAF) and presentation method (opacity) were analyzed. As the timestamp for stimulus onset, the internal measure *rAF before created* was used, and for the response, *event created*, though the results were similar for other timestamps (all data and analysis scripts are available via the accompanying OSF website). RT overestimation was calculated as the difference between the measured and realized RTs. Table 4 shows descriptives of RT overestimations per device and browser. We compared the sizes of the RT overestimations via Welch *t* tests between all pairs of devices and browsers. All differences were statistically significant, except between the iOS browsers. Hence, there were substantial differences in mean RT overestimation between devices and browsers, which were particularly low for iOS and particularly high for MacOS Safari.

**Table 4:** Descriptives of RT overestimations (in milliseconds) per device and browser, for stimuli that were timed via rAF and presented via opacity

OS	Web Browser	Minimum	Maximum	Mean	*SD*
Android	Chrome	46.0	103.5	69.8	7.4
Android	Firefox	44.9	131.4	66.1	7.5
iOS	Chrome	48.3	109.0	57.9	6.7
iOS	Firefox	48.0	98.0	58.0	7.5
iOS	Safari	48.3	96.3	57.6	6.5
MacOS	Chrome	50.1	124.7	95.4	8.1
MacOS	Firefox	50.0	125.5	78.2	16.1
MacOS	Safari	93.0	163.7	132.9	8.1
Windows	Chrome	64.7	70.6	68.5	1.7
Windows	Firefox	49.8	84.9	61.9	5.7

#### Variance of RT overestimation

We compared the variance of RT overestimations via Levene’s tests between all pairs of devices and browsers. Variances did not differ significantly between Android, iOS, Windows Firefox, and MacOS Safari. All other variances were statistically significant, expect between MacOS Chrome and MacOS Safari. Hence, Windows Chrome added the least random noise to the RT measurement, MacOS Firefox added the most, and other devices and browsers added moderate levels.

#### Distribution and quantization of RT measurements

**Fig. 2:**
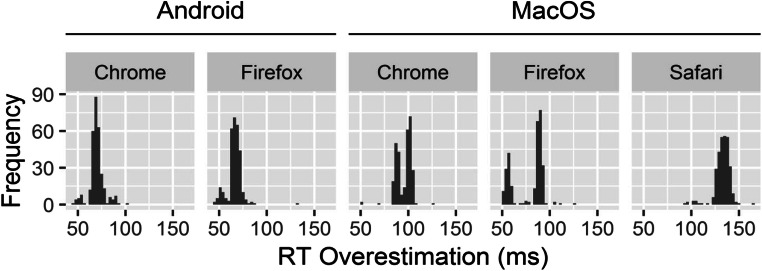
Distributions of response time (RT) overestimations on Android and MacOS.

Inspection of scatterplots of measured RT versus realized RT revealed quantization on iOS with a resolution of 60 Hz. The distributions of RT overestimations for most devices could be described well as having a normal to uniform distribution, with a small number of outliers. However, Android showed some degree of bimodality, whereas MacOS Chrome and MacOS Firefox showed pronounced bimodal distributions (Fig. 2). Fitting a mixture of two normal distributions via expectation maximization (Benaglia, Chauveau, Hunter, & Young, 2009) on RT overestimations in the most extreme case of bimodality (MacOS Firefox) revealed two components, with mean (*SD*) values of 60.0 (12.4) and 89.1 (2.4) ms.

#### Modeling

To examine how the accuracy of RT measurements may affect studies of individual differences, we conducted a set of simulated experiments with 100 participants each. RTs were drawn from an exponentially modified Gaussian distribution. For each simulated participant, the mean of the Gaussian component represented the trait score. The *SD* of the Gaussian component was drawn from a uniform distribution between 25 and 75 ms, and the mean of the exponential component was drawn from a uniform distribution between 50 and 100 ms. To model the accuracy with which devices measured RTs, we added two types of device noise. For each participant, a constant RT overestimation was modeled by adding a constant value to RTs drawn from a uniform distribution between 60 and 130. For each participant, a variable bimodal RT overestimation was modeled by adding to their RTs a value drawn from one of two Gaussian distributions with equal probability. Each Gaussian distribution had an *SD* drawn from a uniform distribution between 2 and 12 ms. The mean of the first Gaussian distribution was 0, whereas the mean of the second Gaussian distribution was drawn from a uniform distribution from 0 to 30 ms.

Two types of experiments were simulated. The absolute-RT experiment consisted of one condition, in which the trait score of a participant was drawn from a univariate Gaussian distribution with *SD*s that varied per experiment. The trait score formed the mean component of that participant’s RTs. The relative-RT experiment consisted of two conditions, with half of the trials belonging to each condition. Two trait scores were drawn from a bivariate Gaussian distribution that was correlated .5 across participants, so both trait scores, as well as their difference, had the same *SD*. Each trait score formed the mean component of that participant’s RT for one of the two conditions. Each experiment was simulated both with and without device noise, with trait *SD*s of 15, 25, and 50 ms, and with trial counts ranging from 10 to 300 in steps of 10. For each of these combinations, 1,000 experiments were simulated. We calculated reliability as the squared Pearson correlation between trait score and mean RT for the absolute-RT experiment, and between trait score difference and relative mean RT for the relative-RT experiment.

**Fig. 3:**
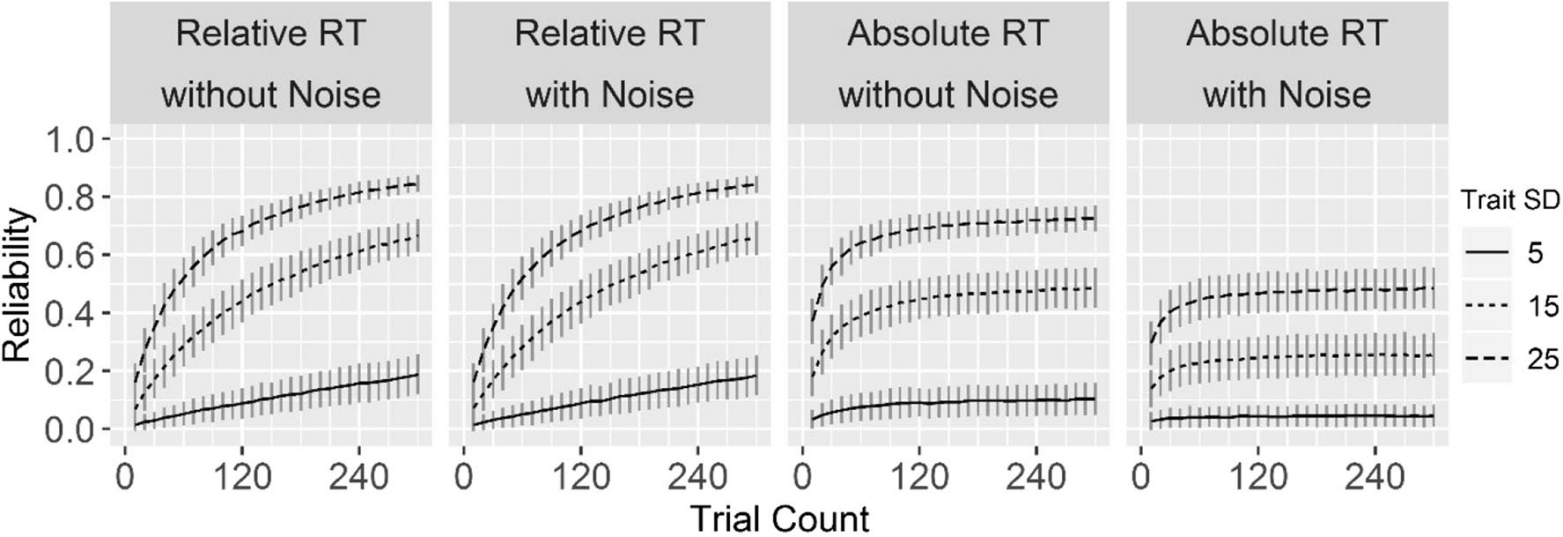
Simulation results for relative and absolute response times (RTs), with and without noise and with trait *SD*s of 15, 25, and 50 ms, for 10 to 300 trials. The lines represent mean reliability, whereas the error bars represent *SD*s.

Figure 3 shows the simulation results. Overall, reliability increased with trial count. The absolute-RT experiments had higher reliabilities than the relative-RT experiments at lower trial counts, but the increase in reliability with trial counts had a smaller slope that leveled off sooner. In the absolute-RT experiments, device noise decreased reliability on average by .19, .21, and .12, for trait *SD*s of 15, 25, and 50 ms, respectively. In the relative-RT experiments, device noise decreased reliability by .012 at most across all trial counts and trait *SD*s. Without noise, the relative-RT experiments were more reliable than the absolute-RT experiments from 120 to 130 trials and beyond. With noise, the relative-RT experiments were more reliable from 40 to 50 trials and beyond.

## Discussion

We examined how accurately web applications on touchscreen and keyboard devices present stimuli for specified durations in Experiment 1, and measured RTs in Experiment 2. In a simulation, we examined how the accuracy of RT measurements affected the reliability with which individual differences could be measured. The results of each experiment are discussed below, followed by a general assessment of the technical capabilities of web applications for mental chronometry.

With regard to stimulus presentation, we first compare the results for different methods for timing and presenting stimuli, followed by an assessment of timing accuracy across devices and browsers. Timing via rAF was more accurate at realizing precise stimulus duration than was timing via CSS animations. In part, this was because iOS timed via CSS consistently presented stimuli for one frame longer than requested. In those cases, requesting slightly shorter durations than was done in this study could improve the accuracy of stimuli timed with CSS animations. However, such consistency was not found for all devices and browsers, so overall we recommend using rAF for timing stimuli. We suspect that the inconsistencies in the behavior of CSS animations may be due to the standards for CSS animations still being a working draft (World Wide Web Consortium, 2018) at the time of this study.

When timing via rAF, presentation method had a relatively small effect on accuracy, with opacity outperforming background position and canvas by up to five percentage points. Because presentation methods were a relatively small factor in timing accuracy, compared with timing methods, a researcher might consider choosing a presentation method on the basis of practical considerations. For instance, canvas may be more suitable than opacity or background position when dynamically generating stimuli. Also, note that a range of other presentation methods is supported by web browsers besides the three methods considered here, such as Scalable Vector Graphics and Web Graphics Library (WebGL; Garaizar, Vadillo, & López-de-Ipiña, 2014a). Future research could establish whether the findings reported here generalize to those presentation methods as well.

Internal chronometry measures of stimulus duration were similarly accurate in estimating stimulus duration as counting the number of frames was at realizing them. This finding is different from prior research (Barnhoorn et al., 2015; Garaizar & Reips, 2018), which may be due to differences in study aims and designs. The present study included a larger variety of devices and browsers and was the first to simultaneously compare timing stimuli by counting frames with estimating stimulus duration via internal measures. We found that for devices and browsers for which stimulus timing by counting frames was near perfect, internal measures of stimulus duration [e.g., JavaScript’s window.performance.now() high-resolution timer] were also near perfect. Conversely, for devices and browsers for which timing was less accurate, internal measures were less accurate as well. Hence, any increase in accuracy attributed to internal duration measures in previous studies may have been because the corresponding devices and browsers were very accurate already.

Although in the present study internal chronometry could not provide any improvements in timing accuracy, internal chronometry may provide more general estimates of timing accuracy in a variety of other ways. For instance, an approach based on the regularity with which software events such as rAF occur (Eichstaedt, 2001) may be useful. Also, internal measures can be important for estimating the refresh rate of a device (Anwyl-Irvine et al., 2019). Although it is beyond the scope of this article, we hope to facilitate such approaches by making all data of the present study available for reanalysis; the URL to the OSF repository containing all materials is listed at the end of this article. Additionally, internal chronometry may identify extreme levels of JavaScript load. A simple way of illustrating the latter is by having a JavaScript application run a never-ending loop. So long as this loop is executing, no other events will take place.

On the basis of the most accurate timing and presentation method found in this study (rAF and opacity), we assessed the accuracy with which keyboard and touchscreen devices can time stimuli. Some devices and browsers, of both touchscreen and keyboard type, achieved near-perfect timing: namely, iOS with Chrome, Firefox, and Safari, as well as Windows with Chrome. Hence, in settings where the device and browser can be controlled, web applications can be as accurate as specialized software. Most devices and browsers achieved most presentation durations within one frame of the requested duration, though MacOS Chrome and Safari tended to present durations of up to six frames (100 ms) too briefly. Hence, when the device and browser cannot be controlled, the reliability and validity of mental chronometry paradigms that require brief presentations may be affected.

With regard to the accuracy of RT measurements, different internal measures for RT gave similar results. Quantization of RT into 60 Hz was found on one device, which may be acceptable (Ulricht & Giray, 1989). RT overestimation varied across devices, similar to what was found in previous research (Neath et al., 2011; Reimers & Stewart, 2015). The range of mean RT overestimations was similar to or smaller than the distributions assumed in various simulation studies with between-group designs (Brand & Bradley, 2012; Reimers & Stewart, 2015; Vadillo & Garaizar, 2016). The iOS device had the lowest mean RT overestimations, whereas MacOS had the highest. Windows in combination with Chrome had the smallest variation of RT overestimation, whereas MacOS again had the highest. In general, when the device that is administering mental chronometry can be controlled, RTs may be measured quite accurately, but not at the level that specialized hardware and software, such as button boxes under Linux, can provide (Stewart, 2006). For particular combinations of devices and browsers, namely MacOS with Chrome and Firefox, RT overestimations were bimodally distributed, with centers that could differ up to 30 ms. Given both the similarity of these RT overestimations to results from previous empirical studies and the robustness reported in simulation studies, we assume that the prior recommendations still apply: A decrease in the reliability of finding group differences in RTs may be compensated for by increasing the number of participants by about 10% (Reimers & Stewart, 2015).

Prior simulations have quantified the impact of the accuracy of RT measurements on the reliability of detecting group differences. As far as we are aware, none have quantified the impact on the reliability of measuring individual differences. Our modeling work indicated that different factors may affect reliability, including the number of trials and the variance of the trait that is measured. The reliability of absolute RT measurements was affected by device noise, but relative RT was hardly affected. This could be because between-device variation was larger than within-device variation. For relative RT, between-device variation is removed due to RTs being subtracted between conditions within participants. A rather striking result was that with higher numbers of trials, relative RT was more reliable than absolute RT, even though traits in the relative RT simulations were correlated .5. The former may appear to go against the commonly held belief that the difference between two positively correlated scores is less reliable than each of these scores individually. Although a comprehensive examination of this result is beyond the scope of this article, here we may offer some explanations. First, a classic result underlying the formerly mentioned belief is based on two observations per participant (Lord & Novick, 1968), but aggregations across larger numbers of observations may yield more reliable difference scores (Miller & Ulrich, 2013). Second, we modeled latent traits as mean and differences between the mean components of ex-Gaussian RT distributions. The distribution of absolute RTs was more skewed than the distribution of relative RTs, so the mean absolute RT was perhaps a less reliable estimator of the trait score than the relative mean RT was of differences in trait scores.

However, in both group and individual difference research, any confound between device type and study design could affect RT results more severely (Reimers & Stewart, 2015). For instance, in a longitudinal study, participants could upgrade their devices between observations. If newer devices have lower RT overestimations, this could result in a spurious decrease in measured absolute RTs over time. Another example of such a confound is when participant traits covary with device preference. Personality research found that Mac users are more open to experience than PC users (Buchanan & Reips, 2001). If Mac overestimates RTs more than PC does, as was found in our sample of devices, this could result in a spurious covariance between openness to experience and an absolute-RT measure. Although more recent studies have shown negligible differences in personality across a number of brands (Gotz, Stieger, & Reips, 2017), similar risks apply to any trait for which covariation with device preference has not been studied. In the case of relative RTs, risks are less severe. Nevertheless, differences between devices with regard to the accuracy with which RT is measured can cause differences in measurement reliability, which in turn can cause violations of measurement invariance.

In summary, in controlled settings, web applications may time stimuli quite accurately and may register RTs sufficiently accurately when a constant overestimation of RTs is acceptable. In uncontrolled settings, web applications may time stimuli insufficiently accurately for mental chronometry paradigms that require brief stimulus presentations. Differences in the degree to which devices overestimate RT may more severely affect the reliability with which individual differences are measured via absolute RT than via relative RT.

Web applications offer a means to deploy studies both inside and outside of the lab. Frameworks are being developed that make it increasingly easier for researchers to deploy mental chronometry paradigms as web applications (Anwyl-Irvine et al., 2019; De Leeuw, 2015; Henninger, Shevchenko, Mertens, Kieslich, & Hilbig, 2019; Murre, 2016). Studies of timing accuracy suggest limits to what may be achieved, but also introduce technical innovations for achieving higher accuracy. The experiments reported in this article examined a range of these technical innovations in order to offer some guidelines on optimal methods. A sample of ten combinations of devices and browsers was studied so that these guidelines and the level of accuracy that can be achieved may be generalized with some confidence.

The results in this study may be representative of web browsers, as the three browsers selected in this study represent a large majority of browsers used online (StatCounter, 2018). However, the sample of four devices was quite small, as compared to the variety of devices available to web applications. This limitation may apply less to MacOS and iOS devices, as their hardware is relatively homogeneous and more to Android and Windows devices, as these come in a very wide range of hardware of different make and quality. Additionally, each included device was a relatively high-end model but was 3–4 years old at the time of the study. Because device technology progresses very rapidly, they may not be representative of newer generations of devices, nor of the budget smartphones that are becoming commonplace in developing countries (Purnell, 2019). Although previous studies reported negligible effects of device load (Barnhoorn et al., 2015; Pinet et al., 2016), so that device load was not included in the present study, this may well be different for such budget smartphones.

A study in a wider range of devices, preferably having multiple samples per device, could replicate the systematic differences found in this study. If replicated, the results could be used to correct for timing inaccuracies and RT overestimations by detecting participants’ device and browser. Note that this undertaking would require significant efforts, given its scale. Also, it would need to be repeated for each new generation of devices, as well as for significant OS and web browser updates. The design of the present study, which could assess timing accuracy at the level of individual trials, could be helpful. By making all materials openly accessible online, we hope to facilitate such efforts.

A solenoid was used for generating responses (similar to Neath et al., 2011). A benefit of the solenoid used in this study was that it provided a method for generating responses that was suitable for both keyboard and touchscreen devices. Responses were defined as the moment the solenoid came in touch with touchscreen or keyboard. Although this is indeed the moment a touch response can be registered, a key needs to be pressed first. Since the actual pressing of a key occurred later than touching it, the registration of responses by the keyboards was only possible to commence at a later point in time than for the touchscreens. However, given the high consistency and speed with which the solenoid went down, we expect this delay to have been 2 ms at most. Given that the RT overestimations we encountered were 57 ms or more, we deem the solenoid-incurred delay to be negligible in light of our findings. Alternatively, keyboard responses could be triggered by disassembling a keyboard and hot-wiring key switches (Pinet et al., 2016; Reimers & Stewart, 2015), and touchscreen responses could be triggered via an electrode (Schatz, Ybarra, & Leitner, 2015).

Overall, touchscreen devices seem technically capable of administering a substantial number of mental chronometry paradigms, when taking some limitations and best practices into account. As smartphone ownership and internet connectivity are becoming ubiquitous, this offers various opportunities for administering mental chronometry on large scales and outside of the lab. By implementing RT tasks as web applications, they are based on durable and open standards, allowing a single implementation to be deployed on desktops, laptops, smartphones, and tablets. We hope that this article helps answer doubts about the timing accuracy of such an approach and provides some insight into how the reliability of RT measurements can be affected when millisecond accuracy cannot be achieved.
